# Adrenal insufficiency in coronavirus disease 2019: a case report

**DOI:** 10.1186/s13256-020-02461-2

**Published:** 2020-08-24

**Authors:** Maryam Heidarpour, Mehrbod Vakhshoori, Saeed Abbasi, Davood Shafie, Nima Rezaei

**Affiliations:** 1 Endocrinology, Isfahan Endocrine and Metabolism Research Center, Isfahan University of Medical Sciences, Isfahan, Iran; 2 Isfahan Cardiovascular Research Center, Cardiovascular Research Institute, Isfahan University of Medical Sciences, Isfahan, Iran; 3 Anesthesiology and Critical Care Research Center, Nosocomial Infection Research Center, Isfahan University of Medical Sciences, Isfahan, Iran; 4 Cardiology/Heart Failure and Transplantation, Heart Failure Research Center, Isfahan Cardiovascular Research Institute, Isfahan University of Medical Sciences, Isfahan, Iran; 5 Research Center for Immunodeficiencies, Children’s Medical Center, Tehran University of Medical Sciences, Tehran, Iran; 6 Department of Immunology, School of Medicine, Tehran University of Medical Sciences, Tehran, Iran; 7 Network of Immunity in Infection, Malignancy and Autoimmunity (NIIMA), Universal Scientific Education and Research Network (USERN), Tehran, Iran

**Keywords:** Adrenal insufficiency, COVID-19, 2019-nCoV, SARS-CoV-2, Case report

## Abstract

**Background:**

Novel coronavirus disease 2019 presents with fever, dry cough, fatigue, and shortness of breath in most cases; however, some rare manifestations in other organs have also been reported so far.

**Case presentation:**

Here, the case of a 69-year-old Iranian man with coronavirus disease 2019 is presented who suffered from frequent episodes of vasopressor-resistant hypotension during intensive care unit admission, which was finally attributed to the occurrence of acute adrenal insufficiency.

**Conclusions:**

As this is a rare complication, adrenal insufficiency might be easily overlooked. However, early detection of this disease among critically ill patients infected with coronavirus disease 2019 could be lifesaving, especially among those unresponsive to vasopressor agents.

## Background

Since late December 2019 to April 2020, more than 2.8 million cases of novel coronavirus disease 2019 (COVID-19) have been reported around the world. COVID-19 has a broad spectrum of severity, ranging from asymptomatic to respiratory distress that requires mechanical ventilation [1–3]**.** However, some patients may develop sepsis, which can happen very quickly and lead to tissue damage, organ failure, and death [2]. In these patients, resistant hypotension might even lead to death in a short time. On the other hand, in the complex setting of critical illness, adrenal insufficiency is easily overlooked as a cause of vasopressor-resistance hypotension [4]. Therefore, it can be assumed that an endocrinological basis could account for some of the extrapulmonary manifestations of COVID-19 infection. Due to the novelty of this pathogen, other presentations of this infection would not be unexpected. In this report, we present the case of a 69-year-old man with COVID-19, who experienced an acute crisis of adrenal insufficiency during hospitalization.

## Case presentation

**Fig. 1:**
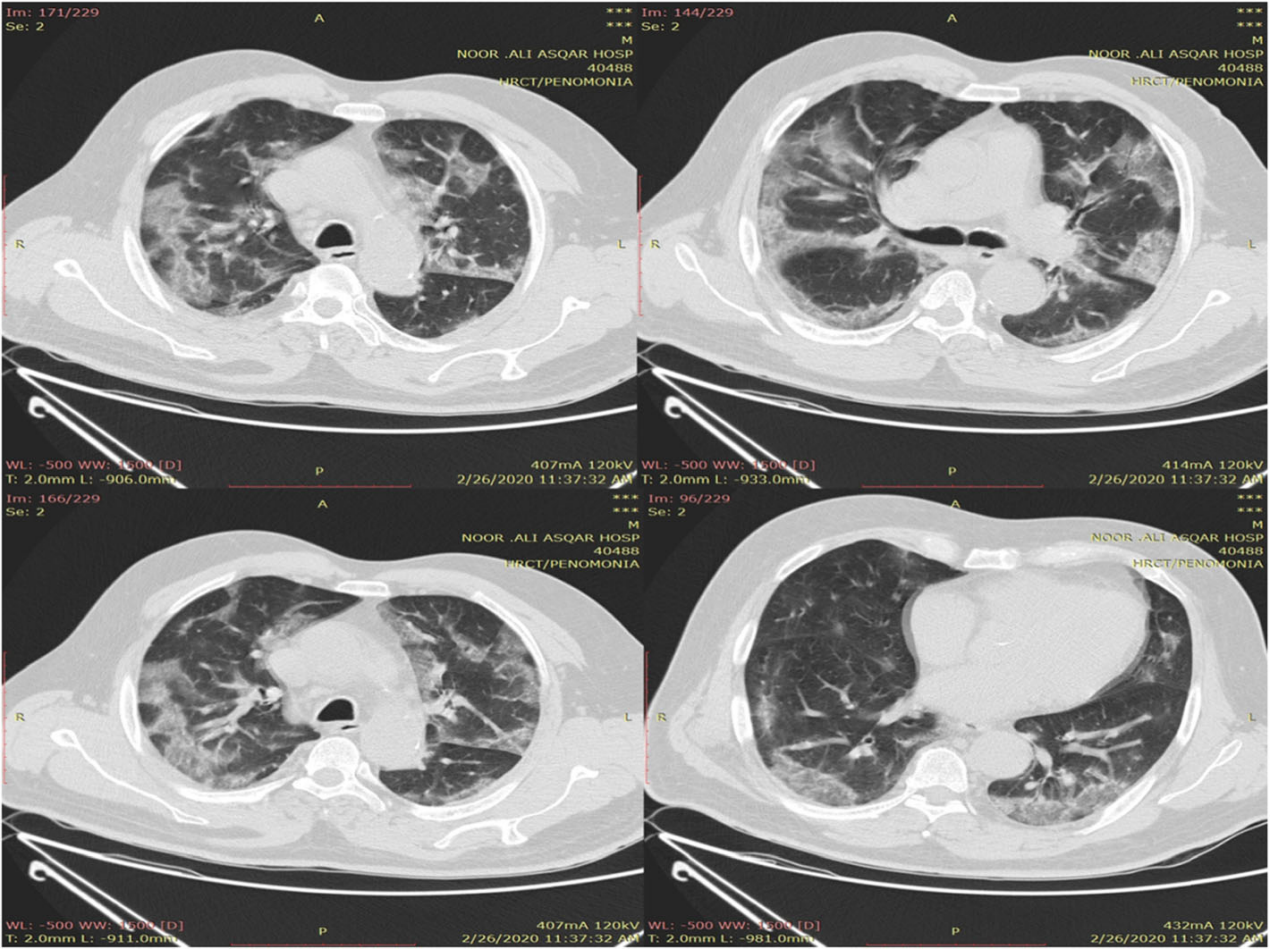
Bilateral and peripheral ground-glass pulmonary opacities

On 27 February 2020, a 69-year-old Iranian man was referred to our hospital, complaining of fever, dyspnea, and dry cough. He had a history of hypertension, which was well controlled with antihypertensive agents. His symptoms started 5 days before admission. Initial vital signs included a blood pressure of 130/80 mmHg, heart rate of 109 beats per minute (bpm), respiratory rate of 28 per minute, O_2_ saturation of 88% on room air, and temperature of 38.3 °C. Laboratory data are shown in Table 1. Due to his respiratory problems, he underwent a chest computed tomography (CT) scan, which showed bilateral and peripheral ground-glass pulmonary opacities suspicious for COVID-19 infection (Fig. 1). His reverse transcriptase-polymerase chain reaction (RT-PCR) test became positive. As a COVID-19 diagnosis was finalized, he was hospitalized in an isolated room. The next day, his dyspnea worsened, and he was intubated and transferred to an intensive care unit (ICU). He received oseltamivir (75 mg every 12 hours) and chloroquine (200 mg every 12 hours), according to the national protocol. However, his fever continued to peak at 39 °C. Blood culture, tracheal aspirate, and urine cultures were taken, and empirical antibiotics were prescribed. His status was complicated on the fifth day with an acute hypotensive episode (systolic blood pressure of 65 mmHg) and diarrhea, which initially responded to fluid resuscitation, but recurred 1 hour later. Despite being on multiple vasopressors and intravenously administered hydration, his blood pressure was consistently low. Blood sugar was within the normal ranges. An electrocardiogram showed sinus tachycardia with no ST-T segment changes. Therefore, as his case was suspicious for adrenal insufficiency, hydrocortisone was administered at a dose of 100 mg, followed by 10 mg per hour administered intravenously after taking a blood sample for a random plasma cortisol level measurement. This method has been suggested to be as effective as a four-dose divided intravenous hydrocortisone prescription [5]. The serum total cortisol level was 12 μg/dl, therefore, no cosyntropin stimulation test was ordered [6]. Intravenously administered hydrocortisone had been administered up to 3 days after the stabilization of our patient’s clinical status in the absence of a vasopressor prescription. His blood pressure remained stable at 110/75 mmHg, and he did not receive any antihypertensive agents because of a prior history of hypertension. He had several further episodes of hypotension in the absence of any vasopressor agent administration during his ICU admission when his corticosteroid regimen was reduced or withheld to perform repeated serum cortisol levels measurement. During his hospitalization, he had three episodes of fever due to nosocomial infections. He was prescribed antibiotics based on the results of blood, tracheal aspirate, and urine cultures. On the 53rd day, his general condition was good, and he received supplementary oxygen via a venturi mask at 40% as well as daily orally administered prednisolone with a dosage of 10 mg.

**Table 1:** Laboratory data of patient at admission and during hospitalization

	At admission	5th day of admission	30th day of admission	53rd day of admission
Hematocrit (%)	42.3	39	36.6	34.5
Hemoglobin (g/dl)	14.1	13	12.2	11.5
White blood cell (cells/μl)	4500	7700	13,800	12,100
Platelets (× 10^6^/l)	142,000	211,000	185,000	188,000
C-reactive protein	Negative (−)	Positive (++)	Positive (+)	Negative (−)
Sodium (mEq/l)	139	135	138	140
Potassium (mEq/l)	3.8	4.1	4.2	3.9
Calcium (mg/dl)	8.7	7.5	7.9	8.5
Phosphorus (mg/dl)	3.1	4.3	3.5	3.2
Albumin (g/dl)	3.5	2	2.9	3.4
Blood urea nitrogen (mg/dl)	24	27	29	26
Creatinine (mg/dl)	1.1	1.2	1.4	0.9
Glucose (mg/dl)	192	125	167	96
International normalized ratio	1.1	1.2	1	1.1
Partial thromboplastin time (seconds)	31	76	32	31
Procalcitonin (ng/ml)	–	0.4	1.1	0.3
Troponin	Negative (−)	Negative (−)	Negative (−)	Negative (−)
Random cortisol (μg/dl)	–	13	15	–
Aspartate aminotransferase (U/l)	40	149	110	31
Alanine aminotransferase (U/l)	31	115	98	28

## Discussion and conclusions

This current report presented an individual infected with COVID-19 with no prior history of adrenal diseases who experienced acute adrenal insufficiency during hospitalization. Due to the new emergence of this virulent pathogen, which mostly affects the respiratory system, other non-respiratory presentations of this infection must be considered, especially in terms of endocrine organs. Adrenal insufficiency seems to be a common problem observed among critically ill patients. It has been reported that patients suffering from severe cases of sepsis, burns, pancreatitis, head trauma, cardiac surgery, or liver disease might experience this disorder. Because of variable definitions and different study populations, the exact incidence of adrenal insufficiency remains unknown [4]. Several mechanisms have been suggested for the development of this disease, including cancer, hemorrhages, thrombosis, autoimmune issues, and drugs, as well as infections leading to sepsis. These aforementioned circumstances may increase cortisol demand, which can result in adrenal insufficiency occurrence.

Moreover, an imbalance between the endocrine system and the immune system might play a role in this regard [4]. The enormous production of inflammatory markers, including interleukin (IL) 1, IL-6, and tumor necrosis factor α (TNF-α), has been suggested to influence the hypothalamic–pituitary–adrenal (HPA) axis because of non-protection of the pituitary gland by the blood–brain barrier. Furthermore, TNF-α leads to decreased adrenocorticotropic hormone (ACTH) release induced by corticotrophin-releasing hormone (CRH) and impairs the function of ACTH and angiotensin 2 on adrenal cells [7–9]. The cytokine storm that resulted from systemic infection caused by COVID-19 might be responsible for the development of adrenal insufficiency. On the other hand, the essential substrate for cortisol production is cholesterol, which is mainly in the form of high-density lipoprotein (HDL). A decrease in HDL level, observed more frequently in severe illnesses, could be considered another possible etiology of adrenal insufficiency [10]. Also, a term named “critical illness-related corticosteroid insufficiency” (CIRCI) has been announced recently. This functional relative adrenal insufficiency is not strictly dependent on cortisol level for diagnosis, but mostly relies on the inadequacy of cortisol for inflammation control or supplying raised metabolic demand [11]. Decreased levels of cortisol carrier proteins, including cortisol-binding globulins (CBGs) or albumin, reduced cortisol-CBG complex cleavage, increased activity of an enzyme responsible for inactivation of cortisol (11-β hydroxysteroid dehydrogenase type 2), as well as decreased numbers of cortisol receptors and affinity have been postulated to be effective in the pathogenesis of this functional syndrome. Overall, because physiological concentrations of corticosteroids play a crucial role in maintaining an appropriate vascular response to vasoconstrictors, adrenal insufficiency was associated with severe resistant hypotension, which was entirely reversed with corticosteroids [5]. On the other hand, it has been suggested that an older member of the *Coronaviridae* family, named severe acute respiratory syndrome (SARS), produces certain amino acid sequences mimicking host ACTH. Consequently, antibody production against this peptide might be responsible for the occurrence of adrenal insufficiency [12]. Moreover, the hypothalamus and pituitary express angiotensin-converting enzyme 2 (ACE2) and SARS genome had been identified in autopsy samples. Therefore, this new coronavirus might also affect the HPA axis and cause acute adrenal insufficiency [13, 14].

In conclusion, adrenal insufficiency should be considered among critically ill patients infected with COVID-19, and high clinical suspicion is required in this regard, especially during hypotensive attacks unresponsive to vasopressor agents. However, the exact etiology for the pathogenesis of this disorder needs to be investigated further.

## Data Availability

The datasets generated during and/or analyzed during the current study are not publicly available due to confidential issues but are available from the corresponding author on reasonable request.

## Abbreviations

COVID-19: Coronavirus disease 2019
ICU: Intensive care unit
bpm: Beats per minute
CT: Computed tomography
RT-PCR: Reverse transcriptase-polymerase chain reaction
IL: Interleukin
TNF-α: Tumor necrosis factor α
HPA: Hypothalamic–pituitary–adrenal
ACTH: Adrenocorticotropic hormone
CRH: Corticotrophin-releasing hormone
HDL: High-density lipoprotein
CIRCI: Critical illness-related corticosteroid insufficiency
CBGs: cortisol-binding globulins
SARS: Severe acute respiratory syndrome
ACE2: Angiotensin-converting enzyme 2
